# When an Overwhelming Number of Supernumerary Teeth Provides an Alternative to the Diagnosis of Trichorhinophalangeal Syndrome

**DOI:** 10.1155/2024/2630240

**Published:** 2024-10-16

**Authors:** Nariman Shaker, Aya Abdelrady, Sara F. A. Haridy, Waleed El-Beialy

**Affiliations:** ^1^Oral Medicine, Periodontology, and Diagnosis Department, Faculty of Oral and Dental Medicine, Future University in Egypt, Cairo, Egypt; ^2^Oral Medicine, Periodontology, and Diagnosis Department at the Faculty of Oral and Dental Medicine, Ain Shams University, Cairo, Egypt; ^3^Oral and Maxillofacial Surgery Department, Faculty of Oral and Dental Medicine, Future University in Egypt, Cairo, Egypt; ^4^Departemnt of Pharmacology, Toxicology and Biochemistry, Faculty of Pharmacy, Future University in Egypt, Cairo, Egypt; ^5^Oral & Maxillofacial Surgery Department, Faculty of Dentistry, Cairo University, Cairo, Egypt

**Keywords:** supernumerary teeth, Trichorhinophalangeal Syndrome Type I, TRPS1

## Abstract

**Background:**

The prevalence of supernumerary teeth is increasing in modern dental practice. However, the presence of multiple supernumerary teeth should be further investigated. Proper diagnosis of an underlying syndrome might save the patient from future health hazards through early diagnosis and optimal follow-up screening.

**Case Presentation:**

A 13-year-old female patient presented with multiple retained deciduous teeth and delayed eruption of permanent teeth. Although the medical and family history of the patient did not raise any concerns, the clinical and radiographic examinations yielded intriguing findings. The patient presented with a total of 11 supernumerary teeth, which impeded the normal eruption of permanent dentition in addition to the presence of retained deciduous teeth. Additional clinical and laboratory investigations were conducted in response to the case's complexity, resulting in the diagnosis of Trichorhinophalangeal Syndrome (TRPS) Type I. The patient underwent a precise treatment plan and then was followed up for 6 months postoperatively to monitor the eruptive movement of the permanent teeth.

**Conclusion:**

When a syndrome is the underlying cause, monitoring unusual cases, such as those with multiple supernumerary teeth, can be lifesaving or aid in the early diagnosis of more serious complications.

## 1. Introduction

Supernumerary teeth, which refer to teeth that exceed the expected number in the primary or permanent dentition, have been found to occur in approximately 0.3%–0.8% and 1.5%–3.5% of deciduous and permanent dentitions [1–3]. Their presence, whether symptomatic or asymptomatic, can result in various complications such as prevention of eruption of permanent teeth, ectopic eruption, crowding, rotation, root resorption of permanent teeth, and pathological follicular cyst formation [4]. Moreover, multiple supernumerary teeth might be indicative of a syndrome with further implications in other areas of the body.

TRPS is one example of a syndrome that can manifest with multiple supernumerary teeth. It is an autosomal dominant genetic syndrome caused by mutation or deletion of the TRPS1 gene of chromosome 8q24 [5]. The phenotype of a typical TRPS patient is characterized by distinctive craniofacial features, ectodermal manifestations, and skeletal abnormalities. TRPS is classified according to the degree of mutation into three subtypes, each with specific landmarks besides the typical phenotype of the syndrome [6]. TRPS1 cases present with short stature and bone deformities. The key features associated with TRPS2 are mental retardation and bone exostoses [7]. The third subtype, known as TRPS3, presents with similar yet more severe manifestations of TRPS1 [8]. There are several reported malformations associated with TRPS, such as those of the genitourinary tract [9, 10] and heart-like ventricular septal defects (VSDs) or mitral valve prolapse (MVP) [11]. Previous studies have linked TRPS with metabolic changes and endocrinological conditions, including hypoglycemia, diabetes, and hypothyroidism. In contrast, other studies reported growth hormone deficiency and bone density changes in patients with TRPS Type I [12].

## 2. Case Presentation

**Figure 1:**
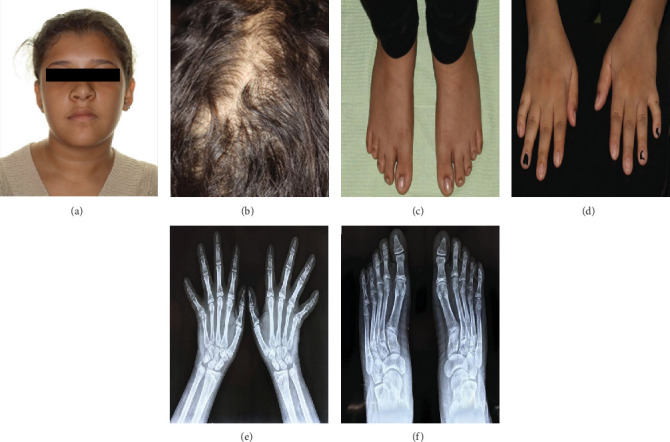
(a) Broad, rounded nasal tip; eyebrows are thicker medially. (b) Thin and sparse hair with areas of the scalp showing. (c) Brachydactyly of the toes. (d) Brachydactyly of the fingers with radial deviation of the distal phalanges of the little finger of both hands. (e) Hand radiography showing brachydactyly, cone-shaped epiphyses, and radial deviation of the little finger. (f) Feet radiography showing brachydactyly of the toes.

The present case involves a 13-year-old female patient complaining of poor esthetics and delayed eruption of her permanent teeth, presented with no family history of supernumerary teeth and no alarming medical history. After conducting a thorough extraoral examination, the following observations were made: a rounded nose tip, scarce hair follicles, and thin hair revealing more areas of the scalp. In contrast, intraoral examination revealed multiple retained deciduous teeth and hard bony expansion of the left mandibular alveolar ridge in the premolar region and the upper right palatal anterior region. The observed extraoral characteristics indicated the possibility of an underlying syndrome. Consequently, additional examination and medical data were collected, revealing the presence of brachydactyly, radial deviation of the little fingers, hyperhidrosis in the hands and feet, and unilateral deficient breast growth (Figure 1).

**Figure 2:**
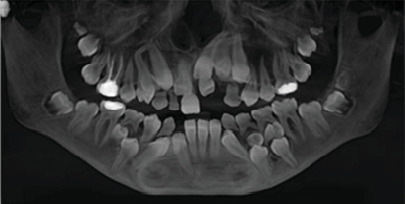
A preoperative OPG showing the supernumerary, permanent, and retained deciduous teeth.

**Figure 3:**
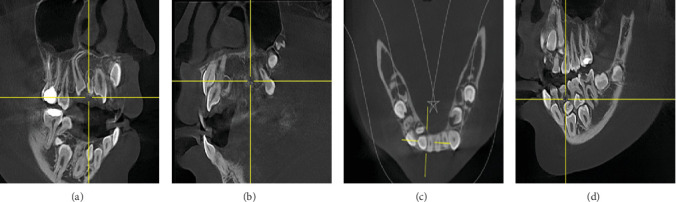
(a) Sagittal cut showing positions of a supernumerary tooth with a root dilaceration and another supernumerary tooth palatal to Tooth 11. (b) Sagittal cut showing the palatal position of ST3. (c) Axial cut of lower right alveolar ridge showing the positions of two supernumerary teeth. (d) Sagittal cut showing the position of another two supernumerary teeth.

The patient underwent radiographic examinations, including a scouting orthopantomogram (OPG), which showed the presence of eight retained deciduous teeth, 10 impacted permanent teeth, and 11 supernumerary teeth (Figure 2). Therefore, a cone-beam computed tomography (CBCT) was carried out for a detailed three-dimensional evaluation, as depicted in Figure 3.

• In the upper right maxillary region: impaction of two supernumerary teeth, the permanent lateral incisor, permanent canine, and the development of a distomolar (Figure 3(a)).
• In the upper left maxillary region: impaction of two supernumerary teeth, the permanent central and lateral incisors, the permanent canine, and the development of a distomolar (Figure 3(b)).
• In the lower-left mandible: impaction of two supernumerary teeth and the two premolars (Figure 3(c)).
• In the lower right mandible: impaction of two supernumerary teeth and the permanent lateral incisor, canine, and second premolar (Figure 3(d)).

### 2.1. Etiology of Supernumerary Teeth Formation

The etiology of the formation of supernumerary teeth is still unclear or relatively contradictory, as several theories have been proposed explaining the occurrence of supernumerary teeth. The aforementioned factors include atavism, the dichotomy of a developing tooth bud, inheritance, and hyperactivity of the dental lamina [13]. The hyperactivity of the dental lamina is the most accepted theory, where the epithelial cord preceding the enamel organ of the permanent tooth does not disappear. It acts as an epithelial remnant that can develop a dental papilla upon stimulation [14].

### 2.2. Differential Diagnosis

The etiology of most supernumerary teeth is idiopathic. However, their presence can be associated with several Mendelian syndromes such as cleidocranial dysplasia, Gardner's syndrome, Anderson–Fabry disease, TRPS Types I and II, Rubinstein–Taybi syndrome, Nance–Horan syndrome, Opitz BBB/G syndrome, and oculofaciocardiodental syndrome. A comparison of the current case to the aforementioned syndromes is depicted in Table 1.

### 2.3. Management of Supernumerary Teeth

**Figure 4:**
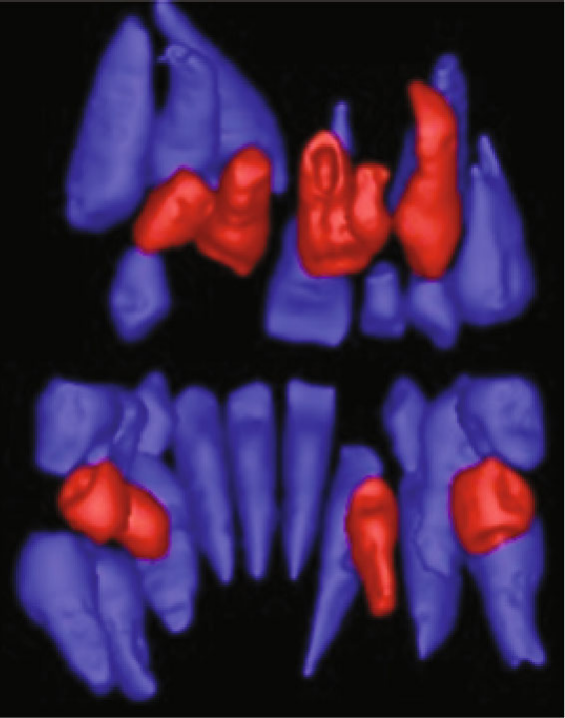
A segmented picture of the preoperative cone-beam computed tomography (CBCT) showing the positions of the nine supernumerary teeth (colored red) from a posterior view.

In the current case, the number of supernumerary teeth, their location within the jaw bones, as well as their association with unerupted permanent dentition, and potential complications involving vital structures necessitated a careful treatment plan. The CBCT was used to evaluate from a three-dimensional perspective (Figure 4). This evaluation aimed to develop a treatment strategy that would facilitate the unobstructed eruption of permanent teeth while avoiding complications such as jaw bone defects, impact on open apices of permanent teeth, damage to nerves and blood vessels, and prevention of maxillary sinus perforation.

**Figure 5:**
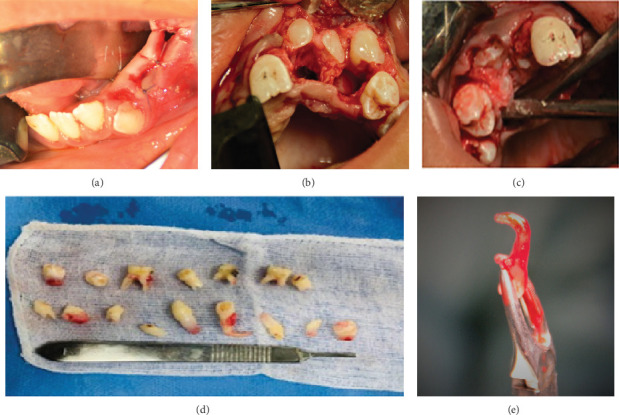
(a) Suturing of interdental papillae only, leaving open sockets to facilitate the eruption of the impacted premolars. (b) Exposure of the impacted permanent anterior teeth to facilitate their eruption by the removal of bone impeding their eruptive movement. (c) Extraction of 52 and 53, followed by flap reflection to expose the supernumerary teeth. (d) Seventeen teeth were extracted. (e) A dilacerated anterior supernumerary tooth.

The treatment plan involved surgical removal under general anesthesia of nine of the 11 supernumerary teeth and eight retained deciduous teeth (Figure 5). When permanent teeth experienced a delayed eruption beyond their expected dates, their eruption paths were cleared from any bony hindrance to facilitate their eruption. Then, the patient was followed up between a 4- and 6-month period to ensure spontaneous eruption of permanents. Subsequently, orthodontic traction may be required to aid traction of unerupted teeth into their normal position and to realign the teeth into proper occlusion.

### 2.4. Follow-Up

**Figure 6:**
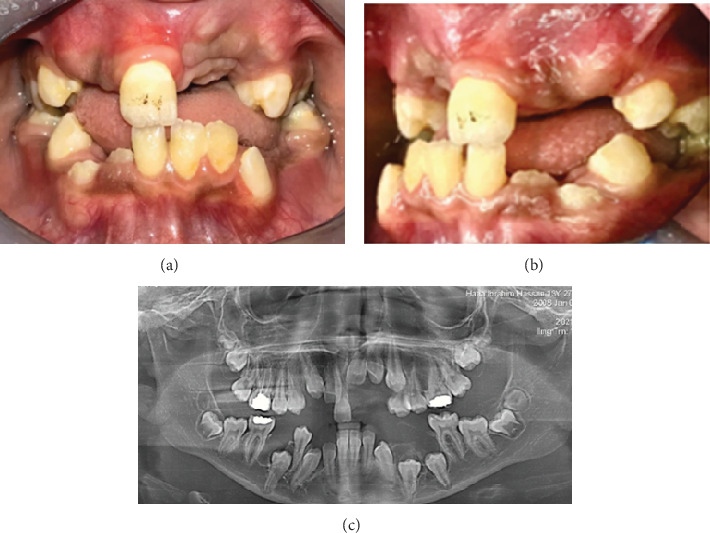
(a, b) One-month postoperative clinical photos: (a) frontal photograph showing eruption of Teeth 42 and 43 and (b) oblique frontal photograph showing eruption of Teeth 42 and 43. (c) Postoperative OPG showing the maxilla and mandible cleared of all supernumerary teeth.

**Figure 7:**
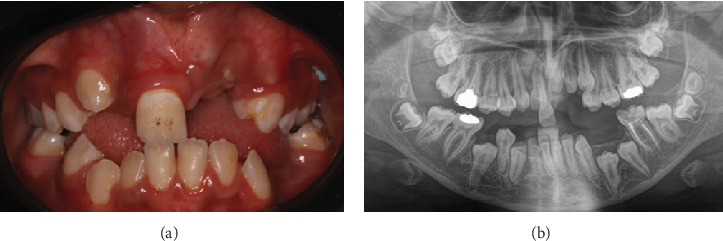
Six months postoperative. (a) Clinical photo demonstrating initial eruption of Teeth 13, 22, and 23. (b) An OPG showing progress in the eruption of the permanent teeth.

The patient was carefully followed up postoperatively to monitor the wound care and oral hygiene measures as well as inspect the progress in permanent dentition eruption. Teeth 42 and 43 became clinically visible 1-month postsurgery (Figure 6). Six months postoperatively, clinical pictures and an OPG were done to determine changes in the positions of the impacted permanent teeth and assess the need for orthodontic intervention (Figure 7).

### 2.5. Genetic Testing

**Figure 8:**
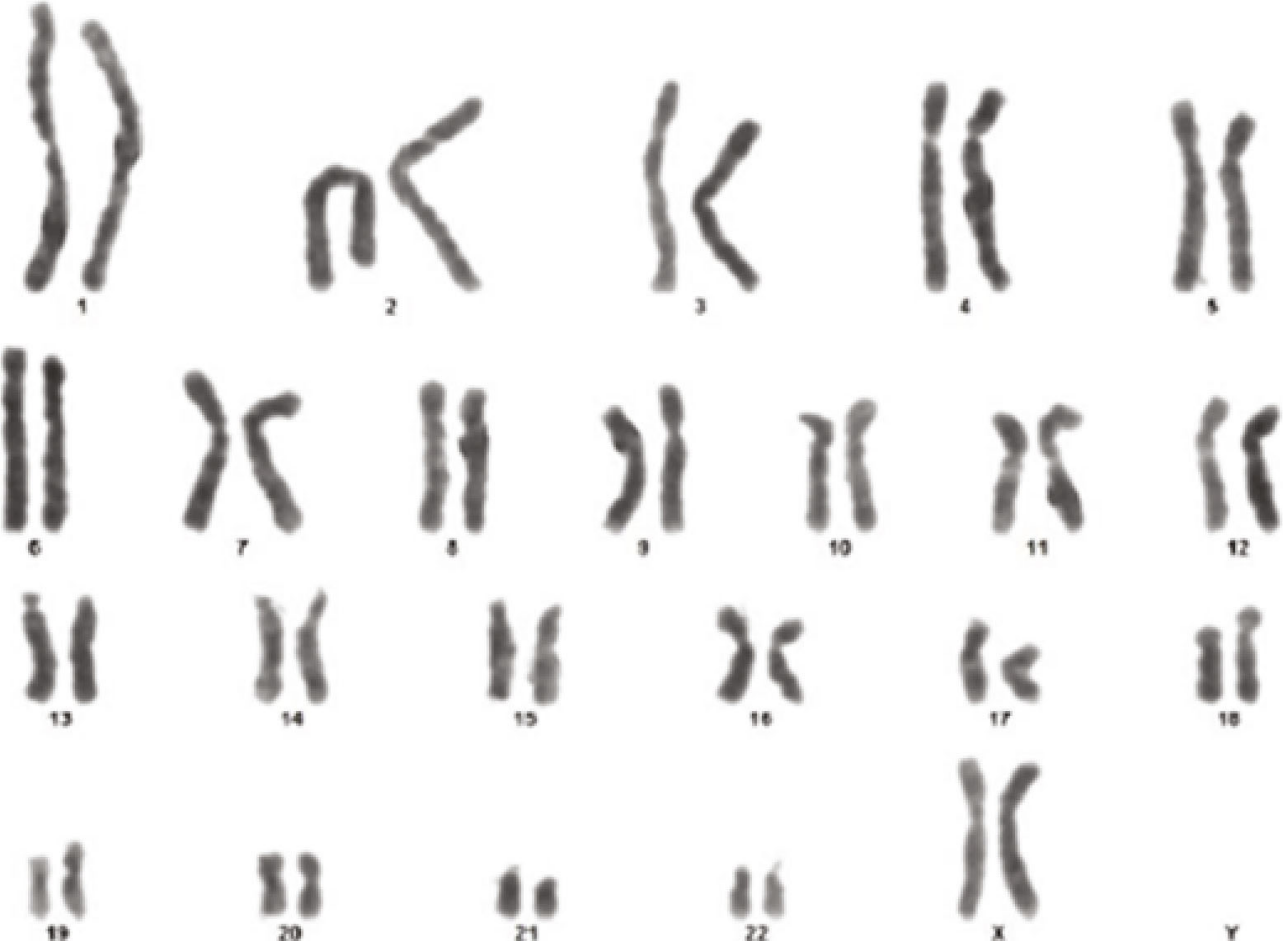
Karyotyping report demonstrating normal chromosomal analysis (46, XX).

Genetic testing plays a crucial role in the diagnosis of syndromes. In the present study, gene testing was performed on the subject by extracting 3 mL of peripheral blood samples from both the patient and her sisters using heparin-containing vaccinator tubes. These samples were then utilized for karyotyping analysis. The results of the chromosomal analysis did not indicate any presence of translocation or deletion. In addition, a normal 46 (XX) karyotype was observed in the case and her siblings (Figure 8), which agrees with the findings of Yamamoto et al., who reported a normal karyotype with typical TRPS Type 1 syndrome [24].

## 3. Discussion

Supernumerary teeth are an odontostomatologic anomaly characterized by the presence of teeth that are additional to the normal series of primary or permanent dentition. The presence of supernumerary teeth can be indicative of an underlying syndrome such as the TRPS [3]. TRPS is an autosomal dominant genetic disorder, first described by Langer–Giedion in 1966, caused by mutations to the TRPS1 gene on Chromosome 8. Giedion named the syndrome based on various craniofacial features and ectodermal and skeletal abnormalities. The clinical features shared by the three subtypes of TRPS include sparse hair, long philtrum, bulbous nasal tip, brachydactyly, cone-shaped epiphyses, and growth impairment [25].

The current case presented had a phenotype almost typical of the TRPS Type I described in the literature [2, 5]. The skeletal features manifested as brachydactyly, cone-shaped phalanges, radial deviation of the pinky finger, and short stature. The ectodermal manifestations observed in the patient included thin, scarce hair, medially thick eyebrows, and the development of 11 supernumerary teeth. The latter manifestation, characterized by the presence of additional teeth, was particularly noteworthy and served as the primary impetus for further investigations. The diagnosis of TRPS is typically established by corroborating the existence of distinctive physical characteristics associated with the syndrome through a comprehensive examination of the patient's medical background, clinical assessment, and radiographic imaging of the skeletal system to confirm any abnormalities in the hands and feet. Despite its classification as a rare autosomal dominant syndrome, the present case and her siblings did not exhibit any indications of inheritance or genetic mutation, consistent with findings reported in previous studies [24, 26]. TRPS manifestations are not limited to the aforementioned skeletal and ectodermal manifestations but involve a wider variety of genitourinary [9], cardiac, metabolic, and endocrinology manifestations [11, 12]. Understanding these potential conditions allows proper screening of multiple systems at such a young age, facilitating the early detection of symptoms and ultimately enhancing the quality of life for the patient.

## 4. Conclusions

The presence of multiple supernumerary teeth in an individual necessitates additional investigation due to the potential association with other manifestations that indicate the presence of an underlying syndrome. The low prevalence rate and clinical phenotype of TRPSI are often overlooked, and the syndrome remains undiagnosed in most of the affected cases. Comprehensive examinations and history recording are crucial for proper diagnosis, followed by regular follow-up visits to monitor changes in the patient's general health, thereby enhancing the patient's quality of life.

## Figures and Tables

**Table 1:** Clinical features of syndromes associated with supernumerary teeth.

**Syndromes associated with multiple supernumerary teeth**	**Clinical features**	**Current case**
Cleidocranial dysplasia [15]	• Delayed closure of the fontanelles • Hypoplastic clavicles • Short stature • Dental abnormalities (retention of primary teeth, delayed eruption of permanent teeth, supernumerary teeth, and multiple impacted teeth)	Absence of all features except dental abnormalities
Gardner's syndrome [16]	• Osteomas • Fibrous dysplasia of the skull • Fibromas • Desmoid tumors • Epidermoid cysts • Supernumerary teeth	Absence of all features except the supernumerary teeth
Trichorhinopharyngeal Syndrome Type I [17]	• Thin scalp hairs, mild diffuse alopecia, and receding frontoparietal hairline	Present
	• Round, pear-shaped nose	Present
	• Elongated philtrum	Absent
	• Thin upper lip	Absent
	• Bone deformities and thin nails (especially cone-shaped epiphyses of the phalanges)	Present
	• Supernumerary teeth	Present
Rubinstein–Taybi syndrome [18]	• Intellectual disability • Short stature • Microcephaly • Broad thumb and halluces • High palate • Unerupted supernumerary teeth and talon cusps	Absence of all features except for the supernumerary teeth
Nance–Horan syndrome [19, 20]	• Craniofacial alterations • Congenital cataracts • Dysmorphic features • Intellectual disabilities • Supernumerary teeth and dental abnormalities	Absence of all features except for the supernumerary teeth
Opitz BBB/G syndrome [21, 22]	• Widely spaced eyes • Laryngo-tracheo-esophageal anomalies • Imperforate anus • Cardiac defects • Oral manifestations: clefting, supernumerary teeth, micrognathia, ankyloglossia, and high-arched palate	Absence of all features except for the supernumerary teeth
Oculofaciocardiodental syndrome [23]	• Microphthalmia • Congenital cataracts • Cardiac symptoms (atrial septal defect and/or ventricular septal defect or mitral valve prolapse) • Facial elongation • High nasal bridge and broad nasal tip with separation of anterior cartilage • Supernumerary teeth and crowding	Absence of all features except for the supernumerary teeth

## Data Availability

The data that support the findings of this study are available on request from the corresponding author.

## Nomenclature

CBCT: cone-beam computed tomography
OPG: orthopantomogram
TRPS: trichorhinophalangeal syndrome
